# Dual-level clustering ensemble algorithm with three consensus strategies

**DOI:** 10.1038/s41598-023-49947-9

**Published:** 2023-12-18

**Authors:** Yunxiao Shan, Shu Li, Fuxiang Li, Yuxin Cui, Minghua Chen

**Affiliations:** 1 School of Science, Harbin University of Science and Technology, Harbin, 150080 China; 2 Key Laboratory of Engineering Dielectric and Applications (Ministry of Education), School of Electrical and Electronic Engineering, Harbin University of Science and Technology, Harbin, 150080 China

**Keywords:** Applied mathematics, Computer science, Information technology

## Abstract

Clustering ensemble (CE), renowned for its robust and potent consensus capability, has garnered significant attention from scholars in recent years and has achieved numerous noteworthy breakthroughs. Nevertheless, three key issues persist: (1) the majority of CE selection strategies rely on preset parameters or empirical knowledge as a premise, lacking adaptive selectivity; (2) the construction of co-association matrix is excessively one-sided; (3) the CE method lacks a more macro perspective to reconcile the conflicts among different consensus results. To address these aforementioned problems, a dual-level clustering ensemble algorithm with three consensus strategies is proposed. Firstly, a backward clustering ensemble selection framework is devised, and its built-in selection strategy can adaptively eliminate redundant members. Then, at the base clustering consensus level, taking into account the interplay between actual spatial location information and the co-occurrence frequency, two modified relation matrices are reconstructed, resulting in the development of two consensus methods with different modes. Additionally, at the CE consensus level with a broader perspective, an adjustable Dempster–Shafer evidence theory is developed as the third consensus method in present algorithm to dynamically fuse multiple ensemble results. Experimental results demonstrate that compared to seven other state-of-the-art and typical CE algorithms, the proposed algorithm exhibits exceptional consensus ability and robustness.

## Introduction

Clustering is an unsupervised analysis technique, which plays a crucial role in exploring the internal structure information of data. Over time, various forms of single clustering methods have been developed. However, the limited scope of application prevents their simultaneous application to datasets with diverse distribution characteristics^1^. Therefore, clustering ensemble (CE) stands out as an extended version of traditional clustering by adopting the concept of ensemble learning. Its objective is to integrate multiple base clustering information in order to generate a final clustering result with enhanced performance, which cannot be achieved by any single clustering method. CE possesses inherent unique advantages in terms of privacy protection and knowledge reuse, thereby avoiding information leakage caused by direct access to the original dataset.

It has been widely confirmed by numerous previous studies that achieving the optimal clustering result through the integration of all member information is not always feasible^2–10^. The presence of members with subpar quality can impede the effectiveness of ensemble methods. In light of this, the emergence of clustering ensemble selection (CES) technology serves as a remedy to mitigate this inherent risk. Nevertheless, it is important to note that there are still several obstacles and challenges that need to be overcome in order to attain a consensus result with better performance within the CES framework.

1. How to select base clustering members adaptively. It is understood that the majority of selection strategies, including soft and hard selection strategies, necessitate the establishment of parameter thresholds or empirical knowledge in advance for their implementation^2–5,11–13^. The algorithm's dependence on parameters and the dataset's structure will intensify, leading to an increase in the time-consuming issue associated with parameter tuning.
2. How to construct a more accurate representation of relationships between sample pairs. Scholars have expended considerable efforts towards reconstructing the relationship matrix, incorporating techniques such as rough set theory, random walk, and dark knowledge, among others. However, the current approaches focus on extracting information from the base clustering results, neglecting the influence of the actual spatial location information between samples. This leads to a one-sided analysis of the relation matrix.
3. How to solve the highly conflicting problem of the inconsistent division at the CE consensus level. As an efficient approach to address conflicts and uncertainties, the traditional Dempster-Shafer (DS) evidence theory has been employed to integrate various base clustering outcomes within the same CE method. At this juncture, an issue is likely to arise. Specifically, the traditional DS evidence theory demonstrates a lack of robustness in instances characterized by a high level of conflict among evidences. This shortcoming could potentially diminish the credibility of the fusion results and further undermine the conflict resolution capabilities of the DS theory. There exists a more significant aspect that this conflict persists at an elevated level, even after the consensus is achieved via diverse CE methods. That is to say, to effectively address the issue of high conflict at the consensus function level, the development of a novel approach presents a significant challenge.

Therefore, the present study proposes a backward clustering ensemble selection framework (BCESF), and designs a dual-level clustering ensemble algorithm with three consensus strategies based on the BCESF. In one aspect, BCESF presents an alternative effective selection strategy for CES that avoids the need for parameter thresholds or human intervention. In another aspect, two consensus strategies, utilizing the relationship matrix as crucial input, are designed at the base clustering consensus level. The co-occurrence frequency and actual spatial location information are simultaneously considered to reconstruct the relation matrices, which facilitate the mining of more realistic data structure information. Additionally, this study employs an enhanced adjustable DS evidence theory for the first time to integrate diverse ensemble results at the CE consensus level, offering a broader perspective. This constitutes the third consensus strategy proposed in this work. This consensus strategy not only adaptively adjusts label probabilities to accommodate changes in dataset structure and integration methods, but also exhibits superior conflict resolution capabilities compared to traditional DS evidence theory, thus facilitating the attainment of higher-confidence consensus results.

## Related works

In this section, we provide a concise overview of the underlying background and theoretical concepts relevant to this study, including a discussion of various effective processing techniques across different stages of CE, alongside DS evidence theory.

### Clustering ensemble

At present, the CE technology has been effectively implemented in various data mining domains, such as bioinformatics, multimedia data analysis, dynamic detection, statistics, social network analysis and pattern recognition, among others^11,14–25^. The process of CES involves selecting base clustering members through an additional step beyond the CE method, prior to achieving consensus. Consequently, the CES implementation framework can be distilled into three processes: (1) base clustering generation process; (2) base clustering members selection process; (3) consensus clustering process. Occasionally, the latter two processes involve interleaving and iteration due to varying selection strategy principles. The treatment of the first and third processes is compatible and can be shared in both frameworks.

In the execution of the first process, the equilibrium between the quality and diversity of base clustering members has consistently been the central point of exploration. Higher quality means that the division of base clusterings is uniform highly, while higher diversity denotes substantial disparities between base clusterings. It is invariably anticipated to extract more knowledge from diverse perspectives to overcome the delusion induced by data deviation. Consequently, a multitude of distinct base clustering generation techniques have emerged. For instance, random initialization method, feature subspace method, multiple single cluster generation methods, resampling method and so forth^26–28^. Obviously, atop these existing technologies, the development of more sophisticated processing methods is indispensable to optimize the overall performance of the algorithm.

In the second implementation process, it is imperative to identify the optimal base clustering members combination adhering to a specific criterion. The objective of this process is to remove the division of relative redundancy and establish a more advantageous information foundation for consensus clustering. The majority of researchers evaluate the combination of base clustering members from the perspective of quality and diversity. Consequently, a series of methods have been proposed. Naldi et al.^11^ employed six distinct criteria to assess the quality of base clustering, and the selected combination of members was used to get the final fusion result. Wang et al.^6^ incorporated rough set theory to screen base clustering subset with a more positive contribution. Lu^7^ and Akbari et al.^10^ constructed the diversity measure matrix based on covariance and pairing-constraint respectively, presenting two novel CES algorithms. Fern and Lin^12^ were the first to employ a comprehensive index derived from the trade-off between quality and diversity for selecting base clustering combinations, and designed three CES strategies. Azimi^13^ and Hong et al.^4^ both proposed the CES method to determine base clustering combination in accordance with preset thresholds. In addition, Hong et al.^4^ used the ensemble results obtained by resampling technique as an index to assess quality and diversity. Zhou et al.^29^ advanced an ensemble learning framework capable of automatically estimating the difficulty of base clustering members and optimize base clustering. Shi et al.^30^ designed a multi-objective self-evolution process to discern the relationship between quality and diversity within the source domain dataset, and facilitate the transfer of this established relationship to the target dataset. Banerjee et al.^31^ introduced a new metric of base clustering quality and diversity, concurrently designing a polynomial heuristic CES algorithm. Khalili et al.^32^ evaluated the diversity/quality of subsets via the Jaccard similarity measure, and adopted three consensus functions to achieve consistent solutions. In addition to this series of evaluation criteria, various strategies from other models were incorporated into the selection process for base clustering members. Yang et al.^33^ optimized the base clustering combination to obtain a CE model with superior performance by integrating the concept of CE with genetic algorithm. Arizad^8^, Nazari^34^, and Parvin et al.^35^ developed multiple strategies with distinct modes for picking class clusters instead of base clusterings. Yu et al.^9^ applied four feature selection methods to obtain the final base clustering subset. Yu et al.^36^ proposed a method that determines the base clustering combination based on the distribution information of various clustering members. In contrast to these hard selection strategies, for example, Li and Ding^37^ introduced a soft selection strategy within NMF framework^38^ to weight base clusterings. However, most CES strategies necessitate the implemented of pre-set parameter thresholds or empirical knowledge, resulting in algorithms that are too dependent on parameters and the structure of the dataset.

When executing the last process, it is crucial to take into account the information of the base clustering members selected by the first two processes, and the corresponding consensus method is employed to generate the final consistent result. From diverse perspectives, numerous effective approaches have been proposed to address the consensus process in this step. Depending on the distinct input information required by the consensus function, consensus methods can be roughly categorized into four types. (1) Graph-based consensus strategy^39–44^. It uses graph theory to partition nodes, thereby generating the final clustering results, in which the nodes are composed of sample points or clusters or both, and the edges represent the relationship between nodes. (2) Co-association matrix-based consensus strategy^45–48^. This kind of method learns the pairwise relationship between sample points by leveraging the base clustering member information, so that the relationship matrix is used as the input for the consensus function to yield the ensemble result. (3) Direct method-based consensus strategy^44,49–51^. This method involves determining the corresponding relationship between clusters, followed by the ultimate division based on voting outcomes. (4) Rough set-based consensus strategy^46,52^. This series of methods analyze the incomplete information generated by different base clusterings, and subsequently obtains the possibility of sample points belonging to a cluster during the final consensus process. Despite the development of numerous consensus strategies, designing those with superior performance remains a significant challenge, particularly at the CE consensus level with a broader perspective.

### Dempster–Shafer evidence theory

The DS evidence theory, initially proposed and refined by mathematicians A. P. Dempster and G. Shafer. As a consequence, an entire suite of evidence theory capable of effectively addressing the uncertainty problem has emerged. In numerous domains of information fusion^52–55^, DS evidence theory has demonstrated its superior ability to resolve conflicts.

According to the description of DS evidence theory, the hypothesis space $\Theta =\left\{{\theta }_{1},{ \theta }_{2},\dots { ,\theta }_{q}\right\}$ is a set of non-empty finite set, which consists of $q$ elements. The power set ${2}^{\Theta }$ of $\Theta$ is defined as Eq. (1):

1 $${2}^{\Theta }= \left\{{A}_{i}|{A}_{i}\subseteq\Theta \right\}$$

The mass function $m$ is a mapping from ${2}^{\Theta }$ to $\left[0, 1\right]$, also known as the basic probability assignment function (BPA) on the hypothesis space. ${A}_{i}$ refers to a specific hypothesis. Then, $m\left({A}_{i}\right)$ represents the probability distribution of hypothesis ${A}_{i}$. Under the conditions of Eqs. (2) and (3), the mass function $m\left({A}_{i}\right)$ is the reliability measure of the final result.

2 $$m\left(\varnothing \right)=0$$

3 $$\sum_{{A}_{i}\subseteq\Theta }m\left({A}_{i}\right)=1$$

where $\varnothing$ is an empty set. Under the premise that the mass function $m$ is known, the definitions of belief function (*Bel*) and plausibility function (*Pl*) are expressed by Eqs. (4) and (5), respectively:

4 $$Bel\left({A}_{i}\right)=\sum_{{B}_{i}\subseteq {A}_{i}}m\left({B}_{i}\right)$$

5 $$Pl\left({A}_{i}\right)=\sum_{{B}_{i}\cap {A}_{i}\ne \mathrm{\varnothing }}m\left({B}_{i}\right)$$

The belief interval $\left[Bel\left({A}_{i}\right), Pl\left({A}_{i}\right)\right]$ represents the degree of confirmation of the hypothesis ${A}_{i}$, and there is a one-to-one correspondence among $m\left({A}_{i}\right)$, $Bel\left({A}_{i}\right)$, and $Pl\left({A}_{i}\right)$. DS evidence theory regards the value with the highest credibility obtained by the fusion rules as the final result. For $\forall A\subseteq\Theta$, the fusion rule of $n$ mass functions $m\left({m}_{1},{ m}_{2},\dots { ,m}_{n}\right)$ is shown in Eq. (6):

6 $$\left({m}_{1}{ \oplus m}_{2} \oplus \dots \oplus { m}_{n}\right)\left(A\right)=\frac{1}{K}\sum_{{A}_{1 }\cap { A}_{2} \cap \cdots \cap { A}_{n}=A}{m}_{1}\left({A}_{1}\right)\cdot {m}_{2}\left({A}_{2}\right)\cdot \dots \cdot {m}_{n}\left({A}_{n}\right)$$

where ${A}_{1},{ A}_{2},\dots { ,A}_{n}\subseteq\Theta$, $K$ is the normalization factor. It represents high degree of conflict when $K$ is 1 or infinitely close to 1. The calculation formula of $K$ is shown in Eq. (7):

7 $$K=\sum_{{A}_{1 }\cap {A}_{2}\cap \cdots \cap {A}_{n}\ne \mathrm{\varnothing }}{m}_{1}\left({A}_{1}\right)\cdot {m}_{2}\left({A}_{2}\right)\cdot \dots \cdot {m}_{n}\left({A}_{n}\right)=1-\sum_{{A}_{1 }\cap {A}_{2}\cap \cdots \cap {A}_{n}=\mathrm{\varnothing }}{m}_{1}\left({A}_{1}\right)\cdot {m}_{2}\left({A}_{2}\right)\cdot \dots \cdot {m}_{n}\left({A}_{n}\right)$$

Precisely because the DS evidence theory can effectively integrate the data and represent uncertain information through its mathematical model without necessitating prior knowledge of the target. This makes it stand out in many fields with conflicting challenges, including the field of CE. Both Wu^53^ and Li^54^ employed DS theory to consolidate multiple single clustering outcomes into a final result, and calculated class probabilities by Gaussian mixture modeling and nearest neighbor techniques, respectively. However, Wang et al.^55^ used the DS theory to fuse multiple clustering validity functions, aiming to identify the optimal number of class clusters. It should be highlighted that the applications of these existing DS evidence theory in CE all focus on the level of fusing single clustering result. Moreover, these approaches consistently employ traditional DS evidence theory to solve the issue. That is, this entails assuming that each clustering result possesses equivalent reliability, disregarding any discrepancies between them. Consequently, when there is a high degree of conflict among different clustering results, the credibility of the final clustering results derived from traditional DS evidence theory will be significantly undermined. Furthermore, it is also unreliable to directly obtain the class probability when the quality of the randomly generated single clustering result is poor, which will still prejudice the final fusion results. As such, this study presents an advanced CE framework from a broader perspective, which can automatically adjust the weight according to the reliability of different CE results at the level of CE consensus, rather than base clustering consensus, thereby minimizing the adverse impact of high conflict.

## Methodology

**Figure 1:**
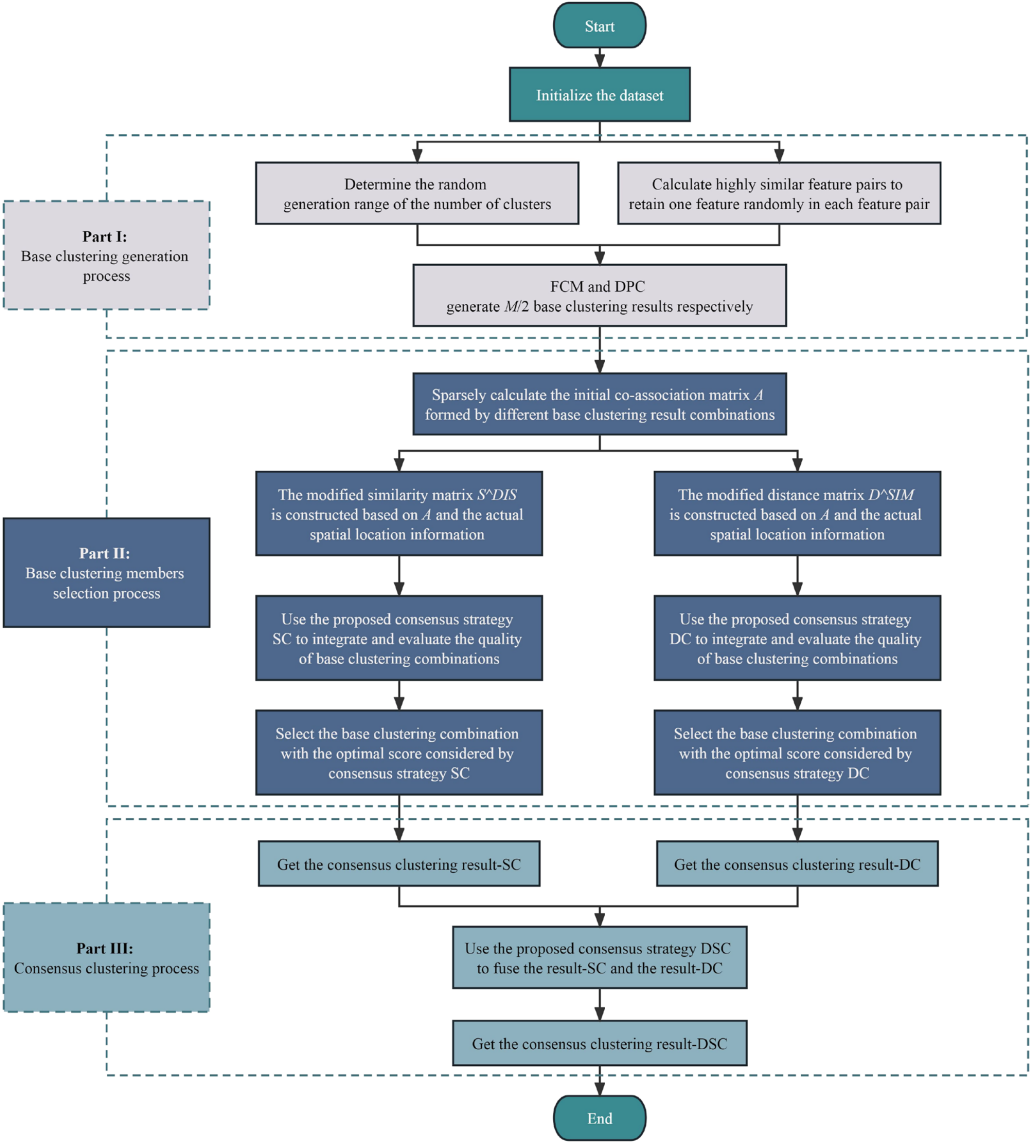
The overall implementation flowchart based on BCESF.

In this section, the proposed backward clustering ensemble selection framework (BCESF) is described in detail. Under this framework, three consensus strategies with varying modes are designed. Specifically, the general form of BCESF is elaborated first; subsequently, two consensus strategies are developed based on two newly defined co-association matrices; ultimately, the third consensus strategy proposed is explicated based on the adjustable DS evidence theory. The overall implementation process facilitated by BCESF is illustrated in Fig. 1.

### Problem formula

The process of CE involves integrating multiple base clustering results to achieve a more ideal consensus result. Generally, the mathematical formulation in the CE problem is defined as follows. Let $X=\left\{{x}_{1},{ x}_{2},\dots { ,x}_{N}\right\}\in {{\text{R}}}^{h}$ denotes a target dataset with $N$ sample points, where ${x}_{i}$ is the $i$ th sample point in the target dataset $X$, and $h$ is the attribute dimension of each sample point. A set of $M$ base clustering results generated by $M$ different parameters or clustering algorithms setting can be described as $\Pi =\left\{{\pi }^{1},{\pi }^{2},\dots ,{\pi }^{M}\right\}$, where ${\pi }^{m}=\left\{{C}_{1}^{m},{C}_{2}^{m},\dots ,{C}_{{a}^{m}}^{m}\right\}$ denotes the $m$ th base clustering in $\Pi$, ${C}_{j}^{m}$ is the $j$ th cluster of the $m$ th base clustering ${\pi }^{m}$, ${a}^{m}$ is the number of clusters in the $m$ th base clustering ${\pi }^{m}$. For convenience, the set of all clusters in the base clustering set $\Pi$ is represented by $C=\left\{{C}_{1},{ C}_{2},\dots { ,C}_{{A}_{C}}\right\}$, ${A}_{C}$ is the total number of all clusters in $\Pi$.

### General form of the backward clustering ensemble selection framework

BCESF can be decomposed into three modules: (a) base clustering generation process; (b) base clustering member subsets selection process and (c) consensus clustering process. In this section, an overview of the implementation mechanism of BCESF is provided, with the specific operational details of each module delineated below.

#### Base clustering generation process

To generate base clustering randomly with better quality and diversity balance, a more refined generation approach is implemented. For the target dataset $X=\left\{{x}_{1},{ x}_{2},\dots { ,x}_{N}\right\}\in {{\text{R}}}^{h}$, $M/2$ base clustering results are generated by fuzzy C-means (FCM)^56^ and density peaks clustering (DPC)^57^ algorithms, respectively. Consequently, a total of $M$ base clustering results $\Pi =\left\{{\pi }^{1},{\pi }^{2},\dots ,{\pi }^{M}\right\}$ are obtained. FCM is a soft clustering method, which accomplishes clustering by updating the membership matrix and the cluster centers. Compared to the K-means algorithm, FCM possesses a stronger ability to handle uncertain points. Meanwhile, DPC is an advanced density-based clustering algorithm that has been intensively studied in recent years. It is suitable for a wider range of data structures^58–60^. Generating base clustering under these two distinct and complementary partitioning methods can better achieve a balance of quality and diversity.

It is worth noting that when the random generation range of the number of clusters is set in the traditional way $\left( {\left[ {2,\left\lfloor {\sqrt N } \right\rfloor } \right]} \right)$, it may generate base clustering that deviate significantly from the actual, especially if the right boundary significantly deviates from the actual number of clusters. Therefore, it is imperative to establish a more plausible right boundary value under the premise of ensuring quality and diversity. To achieve this, we adopt the idea of the DPC algorithm for cluster number screening, enabling the identification of the appropriate right boundary for the random generation range. The identification of cluster centers via the DPC algorithm hinges on two crucial variables. One is the local density ${\rho }_{i}$ and the other is the relative distance ${\delta }_{i}$.

Specifically, the calculation formula of the local density ${\rho }_{i}$ is as follows:

8 $${\rho }_{i}=\sum_{j\ne i}\chi \left({d}_{i,j}-{d}_{c}\right), \chi \left({\text{a}}\right)=\left\{\begin{array}{c}1, a<0\\ 0, a\ge 0\end{array}\right.$$

where ${d}_{c}$ is the cut-off distance, which is regarded as the only hyper-parameter for the consensus strategy.

When ${x}_{i}$ is a non-maximum local density point, relative distance ${\delta }_{i}$ is determined by the nearest sample point ${x}_{j}$, which has a large local density relatively:

9 $${\delta }_{i}={{\text{min}}}_{j:{\rho }_{j}>{\rho }_{i}}\left({d}_{i,j}\right)$$

When ${x}_{i}$ is the maximum local density point, its ${\delta }_{i}$ is denoted as ${\delta }_{{\text{max}}}$, as follows:

10 $${\delta }_{{\text{max}}}={{\text{max}}}_{j}\left({d}_{i,j}\right)$$

The DPC algorithm posits that the cluster centers exhibit two distinct features: greater local density $\rho$ compared with surrounding points, and greater relative $\delta$ distance between cluster centers. Thus, the potential number of candidate cluster centers can be ascertained by leveraging these two salient features and their corresponding expansion processing. The objective of this approach is to lock in a more plausible range of cluster number generation, thereby precluding the emergence of untenable base clustering outcomes which could negatively impact on the final ensemble results. Here, we use ${\rho }_{i}$ and ${\delta }_{i}$ to determine the set ${C}_{P}$ of possible candidate cluster centers:

11 $${C}_{P}=\left\{{x}_{t}|{\rho }_{t}>\frac{\sum_{i=1}^{N}{\rho }_{i}}{N} \&{ \delta }_{t}>\frac{\sum_{i=1}^{N}{\delta }_{i}}{N}, t\in \left[1, N\right]\right\}$$

In fact, any sample point ${x}_{t}$ corresponds to a pair of ${\rho }_{t}$ and ${\delta }_{t}$. Set ${C}_{P}$ stores all sample points where both ${\rho }_{i}$ and ${\delta }_{i}$ are greater than the mean. This means that the real number of clusters will be derived from the potential number of samples in ${C}_{P}$. ${C}_{P}$ provides a reasonable space that closely adheres to reality and inclusive for the random generation range of cluster number. In practical problems, the real number of clusters of a dataset is generally less than $\left|{C}_{P}\right|$ or $\left\lfloor {\sqrt N } \right\rfloor$. Then, the random initialization range of the number of clusters is finally set to $\left[ {2,\min \left( {\left| {C_{P} } \right|,\left\lfloor {\sqrt N } \right\rfloor } \right)} \right]$, where $\left|{C}_{P}\right|$ indicates the number of elements in the set ${C}_{P}$.

In addition, the Pearson correlation coefficient (Pcc) is employed to randomly eliminate one of the attribute pairs characterized by a high degree of correlation, where the attribute pair satisfies $\left|corr( .)\right|>\theta$. The remaining feature attributes are used to generate base clustering results.

By executing the aforementioned process, the generation process of base clustering not only mitigates the emergence of extreme members but also optimally balances quality and diversity. Concurrently, a solid foundation is established for the subsequent execution of crucial steps.

#### Base clustering member subsets selection process

Numerous studies have confirmed that fusing all base clustering information may not necessarily yield the optimal consensus results^26–35^. The involvement of noise members can attenuate the overall ensemble effect. Moreover, most selection strategies necessitate the introduction of additional hyper-parameters as a cost. Consequently, it is crucial to eliminate noise members to enhance the consensus quality of the base clustering combination. In the proposed BCESF, the given consensus function is embedded into the selection strategy and the final base clustering combination is determined by iteration. In theory, any known consensus function can be embedded into the framework for use. The specific execution steps are as follows:

**Step 1:** Starting from the combination $\Pi =\left\{{\pi }^{1},{\pi }^{2},\dots ,{\pi }^{M}\right\}$ composed of all $M$ base clustering results, calculate the Davies–Bouldin index (DBI)^61^ value of the consensus result of the combination under the given consensus strategy. The DBI is defined as follows:

12 $$DBI = \frac{1}{C}\sum\limits_{i = 1}^{C} {\mathop {\max }\limits_{j \ne i} } \left( {\frac{{\overline{{s_{i} }} + \overline{{s_{j} }} }}{{d_{ij} }}} \right)$$

where $C$ is the number of clusters in the consensus result; $\overline{{s_{i} }}$ represents the average distance from the sample points to the center of mass in the $i$ th cluster; ${d}_{ij}$ is the Euclidean distance between the center of mass of cluster $i$ and the center of mass of cluster $j$.

**Step 2:** On the basis of the combination $\Pi =\left\{{\pi }^{1},{\pi }^{2},\dots ,{\pi }^{M}\right\}$ in the previous step, separately calculate the DBI value of $M$ combinations formed by removing one base clustering result in order (each base clustering combination contains $M-1$ base clustering results), and then remove the base clustering from $\Pi =\left\{{\pi }^{1},{\pi }^{2},\dots ,{\pi }^{M}\right\}$ that makes the DBI value of the updated base clustering combination reach the optimal one.

**Step 3:** On the basis of the combination obtained in the previous step, continue to calculate the DBI value independently of $M-1$ combinations formed by removing one base clustering result in order (each base clustering combination contains $M-2$ base clustering results), the base clustering whose DBI value of the updated base clustering combination reaches the optimal will be eliminated. And so on, until there is no base clustering result that can be eliminated.

At this time, the base clustering combination ${\Pi }^{*}=\left\{{\pi }^{1*},{\pi }^{2*},\dots ,{\pi }^{L*}\right\}, L\le M$ with the lowest DBI score is regarded as the best base clustering result subset. So the final consensus result based on ${\Pi }^{*}$ is obtained under the premise of a given consensus strategy.

#### Consensus clustering process

This section delineates the last process of BCESF. Aiming at the execution of this process, three innovative consensus strategies with varying modalities are developed, which are spectral-based (SC), density-peaks-based (DC), and DS-based (DSC). The BCESF-SC is elaborated in the “1) **BCESF-SC**” section, and it belongs to consensus mode which utilizes a modified similarity matrix as input for complete clustering. The BCESF-DC is detailed in the “2) **BCESF-DC**” section, which belongs to consensus mode that employs a modified distance matrix for input to achieve clustering. The third consensus strategy, BCESF-DSC takes class probability matrix as input to accomplish fusion, which is introduced in the “3) **BCESF-DSC**” section. The first two consensus strategies, SC and DC, are executed at the base clustering consensus level by means of a newly defined relationship matrix. However, the third consensus strategy, DSC is founded on the adjustable DS theory, which various different ensemble results at the CE consensus level with a broader perspective.

To mirror the resemblance between sample pairs, the conventional co-association matrix is typically employed as the input of the consensus function in the CES problem. For a given set of base clustering members $\Pi =\left\{{\pi }^{1},{\pi }^{2},\dots ,{\pi }^{M}\right\}$, the set of clusters of all base clustering results in $\Pi$ is $C=\left\{{C}_{1},{ C}_{2},\dots { ,C}_{{A}_{C}}\right\}$. The co-association matrix ${A\in {\mathbb{R}}}_{N\times N}$ represents the degree of similarity between any two samples, and ${a}_{ij}{=\left[A\right]}_{ij}$ denotes the element at $i$-th row $j$-th column of matrix $A$. The larger ${a}_{ij}$ is, the sample points ${x}_{i}$ and ${x}_{j}$ are divided into the same cluster in more base clustering results, which is defined as follows:

13 $${a}_{ij}{=\left[A\right]}_{ij}=\frac{1}{M}\sum_{m=1}^{M}{\delta }_{ij}^{m}$$

14 $${\delta }_{ij}^{m}=\left\{\begin{array}{ll}1, & if {x}_{i},{x}_{j}\in {C}_{r}, r=\mathrm{1,2},\dots ,{A}_{C}\\ 0, & otherwise\end{array}\right.$$

Although the CES algorithm based on traditional co-association matrix addresses numerous practical issues. But in essence, it merely tallies the co-occurrence of sample pairs within each base clustering, neglecting the intrinsic attractiveness disparities between sample pairs. Even within the same cluster, the actual distance has a non-negligible impact on the similarity degree between sample pairs. In view of this, two modified relationship matrices are designed to capture the co-occurrence relationship between sample pairs in a more comprehensive manner. Both matrices concurrently consider the interaction between co-occurrence frequency and local spatial location information. One is the modified similarity matrix ${S}^{DIS}{\in {\mathbb{R}}}_{N\times N}$, which is obtained by modifying the co-association matrix with local spatial location information, and its expression is as follows:

15 $${s}_{ij}^{DIS}{=\left[{S}^{DIS}\right]}_{ij}=\frac{1-{d}_{i,j}^{*}}{2M}\sum_{m=1}^{M}{\delta }_{ij}^{m}$$

16 $${d}_{i,j}^{*}=\frac{{d}_{i,j}-{\text{min}}\left(d\right)}{{\text{max}}\left(d\right)-{\text{min}}\left(d\right)}$$

where ${d}_{i,j}$ is expressed as the Euclidean distance between sample points ${x}_{i}$ and ${x}_{j}$ in the whole text, and it is also the element at $i$-th row $j$-th column of matrix ${D}^{*}{\in {\mathbb{R}}}_{N\times N}$. ${\text{min}}\left(d\right)$ and ${\text{max}}\left(d\right)$ are the minimum and maximum distance values among all distances, respectively.

The other is the modified distance matrix ${D}^{SIM}{\in {\mathbb{R}}}_{N\times N}$, which is derived by modifying the local spatial location information with co-association matrix, and its calculation formula is as follows:

17 $${d}_{ij}^{SIM}={\left[{D}^{SIM}\right]}_{ij}=\frac{M\cdot {d}_{i,j}}{1+\sum_{m=1}^{M}{\delta }_{ij}^{m}}$$

By meticulously examining the two newly constructed relationship matrices, it becomes evident that they not only reflect the co-occurrence relationship of sample pairs in the macro view, but also take into account the intrinsic structure information of the sample pair from a microscopic viewpoint. Both of them are deeply intertwined. The two matrices furnish more precise and realistic input information for the subsequent consensus strategy, enhancing its diversification from a methodological perspective.

##### BCESF-SC

In the consensus strategy of BCESF-SC, a new undirected graph needs to be constructed. Subsequently, the final consensus result is obtained by partitioning the graph, where the sample points are treated as nodes within the graph, and the modified similarity matrix ${S}^{DIS}$ is used as the adjacency matrix between nodes. That is:

18 $$\widetilde{G}=\left(V, \widetilde{E}\right)$$

where $V=X$ is the node set composed of sample points, and $\widetilde{E}$ is the edge set. In the graph $\widetilde{G}$, the edge weights are determined by the modified similarity matrix ${S}^{DIS}$. For a given node ${x}_{i}$ and ${x}_{j}$, the edge weight between them is defined as:

19 $${\widetilde{e}}_{ij}={s}_{ij}^{DIS}$$

Then, the Laplacian matrix of the graph is normalized, which is:

20 $${\widetilde{L}}^{{\text{sym}}}=I-{D}^{-1/2}{S}^{DIS}{D}^{-1/2}$$

where $I$ is the identity matrix. $D\in {{\text{R}}}^{N\times N}$ is a degree matrix with any element on its diagonal ${d}_{i}=\sum_{j=1}^{N}{s}_{ij}^{DIS}$. Next, the eigenvalue decomposition of ${\widetilde{L}}^{{\text{sym}}}$ is performed to obtain the eigenvectors corresponding to the smallest first ${C}^{*}$ eigenvalues. The ${C}^{*}$ eigenvectors are expanded by column normalization to form a new matrix representing $F\in {{\text{R}}}^{N\times {C}^{*}}$. Finally, on the basis of $F$, the K-means clustering algorithm is used to obtain the final consensus clustering result ${\pi }_{SC}$:

21 $${\pi }_{SC}=\mathrm{ BCESF}-{\text{SC}}\left({\Pi }_{SC}^{*}\right)$$

where ${\Pi }_{SC}^{*}$ is the optimal base clustering member combination obtained by embedding SC as a consensus strategy into BCESF. BCESF-SC model can be summarized in Algorithm 1.

##### BCESF-DC

In the consensus strategy of BCESF-DC, the distance matrix between sample points is employed as the input. Two crucial variables, namely the local density ${\rho }_{i}$ and the relative distance ${\delta }_{i}$, are derived based on the distance matrix. The specific calculation methods of ${\rho }_{i}$ and ${\delta }_{i}$ are shown in Eqs. (8)–(10). Then, the cluster centers required by the model are selected according to ${\rho }_{i}$ and ${\delta }_{i}$. Eventually, the remaining non-center points are allocated to achieve the final consensus clustering result. In particular, the modified distance matrix ${D}^{SIM}$ is regarded as the input matrix of the model. That is, ${d}_{ij}^{SIM}$ replaces the fundamental distance information ${d}_{i,j}$ required in the original ${\rho }_{i}$ and ${\delta }_{i}$ calculation formulas, thereby supplementing more similarity information to accurately depict the actual relationship between sample points.

Here, a two-dimensional decision graph is constructed with ${\rho }_{i}$ and ${\delta }_{i}$ as abscissa and ordinate. All the sample points are mapped to the decision graph. Subsequently, the points in the upper right corner of the decision graph are identified as the cluster centers, which have large ${\rho }_{i}$ and ${\delta }_{i}$ relatively. Finally, each remaining non-center point is assigned to the same cluster as its nearest point, which has a larger local density. So far, the final consensus clustering result ${\pi }_{DC}$ is obtained:

22 $${\pi }_{DC}={\text{BCESF}}-{\text{DC}}\left({\Pi }_{DC}^{*}\right)$$

where ${\Pi }_{DC}^{*}$ is the optimal base clustering member combination obtained by embedding DC as a consensus strategy into BCESF. The execution steps of BCESF-DC model are summarized as Algorithm 2.


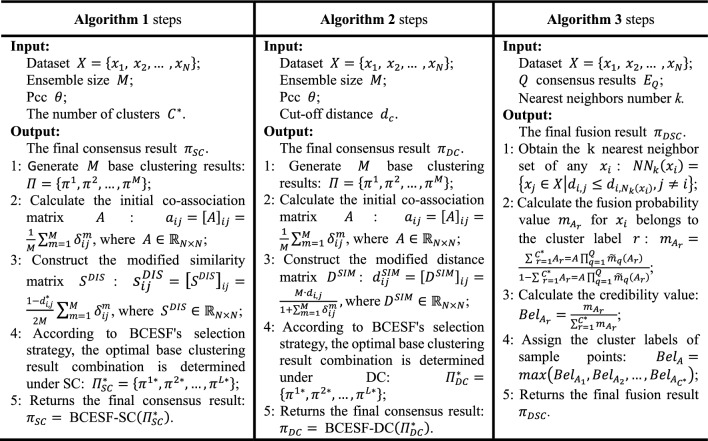


##### BCESF-DSC

**Figure 2:**
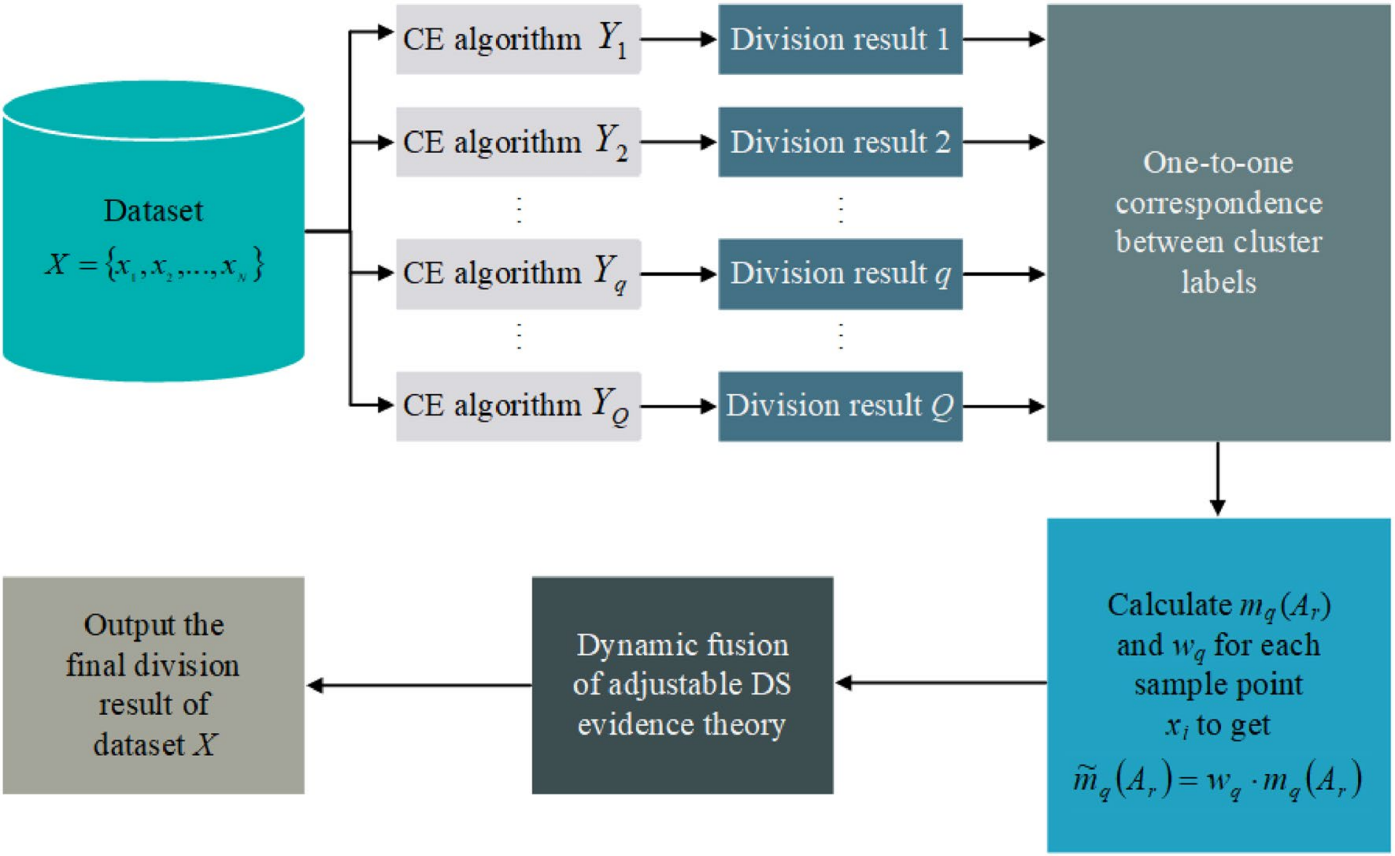
Schematic diagram of adjustable DS evidence theory model.

Following the processing of the target dataset by diverse CE algorithms, the issue of inconsistent partition outcomes remains. That is, there is a lack of a higher-dimensional perspective for globally integrating diverse consistent results. Therefore, we propose a consensus strategy founded on the adjustable DS evidence theory, which effectively addresses the conflicts and contradictions among various consensus results. The fusion diagram of this model is depicted in Fig. 2.

It is worth noting that BCESF-DSC requires that the consensus results derived from distinct CE algorithms all have the same number of clusters ${C}^{*}$. Furthermore, BCESF-DSC matches the cluster labels in various results one by one through the maximum intersection method among clusters. Based on this, the consensus strategy of BCESF-DSC firstly calculates the $k$ nearest neighbors ${NN}_{k}\left({x}_{i}\right)$ of each sample point ${x}_{i}$. The distance matrix required for the calculation process is already obtained when the base clustering outcomes are generated. ${NN}_{k}\left({x}_{i}\right)$ is defined as follows:

23 $${NN}_{k}\left({x}_{i}\right)=\left\{{x}_{j}\in X\left|{d}_{i,j}\le {d}_{i,{N}_{k}\left({x}_{i}\right)}, j\ne i\right.\right\}$$

where ${N}_{k}\left({x}_{i}\right)$ is the $k$-th nearest neighbor for sample point ${x}_{i}$.

Then, an initial ${m}_{q}\left({A}_{r}\right)$ is calculated based on ${NN}_{k}\left({x}_{i}\right)$ and the $q$-th CE algorithm ${Y}_{q}$. ${m}_{q}\left({A}_{r}\right)$ is the basic probability value that the sample point ${x}_{i}$ belongs to the cluster label $r$, and its calculation formula is as follows:

24 $${m}_{q}\left({A}_{r}\right)=\frac{\left|r\left({x}_{j}\right)\right|+1}{{C}^{*}+k}, {x}_{j}\in {NN}_{k}\left({x}_{i}\right) and 1\le r\le {C}^{*}$$

where $\left|r\left({x}_{j}\right)\right|$ represents the number of elements belonging to the cluster label $r$ in the $k$ nearest neighbors of the sample point ${x}_{i}$. Obviously, ${m}_{q}\left({A}_{r}\right)$ can effectively represent the basic probability that any sample point belongs to any cluster label by counting the label distribution in the nearest neighbors.

To minimize the adverse effects induced by high collision, the discrepancies in performance among various CE algorithms are taken into account, and the initial ${m}_{q}\left({A}_{r}\right)$ is updated to ${\widetilde{m}}_{q}\left({A}_{r}\right)$. ${\widetilde{m}}_{q}\left({A}_{r}\right)$ is determined by the adjustable coefficients ${w}_{q}$ and ${m}_{q}\left({A}_{r}\right)$. The expression of the adjustable coefficient ${w}_{q}$ is:

25 $${w}_{q}=\frac{{U}_{q}}{\sum_{q=1}^{Q}{U}_{q}}, q=1, 2,\dots ,Q$$

26 $${U}_{q}=\sqrt{{\sum }_{r=1}^{{C}^{*}}{\left({m}_{q}\left({A}_{r}\right)-1/{C}^{*}\right)}^{2}}$$

Then, ${\widetilde{m}}_{q}\left({A}_{r}\right)$ determined by ${w}_{q}$ and ${m}_{q}\left({A}_{r}\right)$ is described as:

27 $${\widetilde{m}}_{q}\left({A}_{r}\right)={w}_{q}{\cdot m}_{q}\left({A}_{r}\right)$$

Next, the $Q$ consensus results ${E}_{Q}$ can be fused, and the specific fusion result ${m}_{{A}_{r}}$ is shown in Eq. (28):

28 $${m}_{{A}_{r}}=\frac{\sum {}_{r=1}{}^{{C}^{* }}{A}_{r}=A{\prod }_{q=1}^{Q}{\widetilde{m}}_{q}\left({A}_{r}\right)}{1-\sum {}_{r=1}{}^{{C}^{* }}{A}_{r}=A{\prod }_{q=1}^{Q}{\widetilde{m}}_{q}\left({A}_{r}\right)}$$

At this point, the credibility value (*Bel*) of ${A}_{r}$ can be obtained by Eq. (29) :

29 $${Bel}_{{A}_{r}}=\frac{{m}_{{A}_{r}}}{{\sum }_{r=1}^{{C}^{*}}{m}_{{A}_{r}}}$$

Finally, the allocation of cluster labels for sample points is ultimately determined by their calculated credibility values. Specifically, the cluster label with the highest credibility value is the cluster in which the sample point ${x}_{i}$ is located, as illustrated in Eq. (30):

30 $${Bel}_{A}={\text{max}}\left({Bel}_{{A}_{1}}, {Bel}_{{A}_{2}},\dots ,{Bel}_{{A}_{{C}^{*}}}\right)$$

So far, the fusion result ${\pi }_{DSC}$ based on the third consensus strategy DSC is obtained:

31 $${\pi }_{DSC}={\text{BCESF}}-{\text{DSC}}\left({Y}_{1}, {Y}_{2},\dots ,{Y}_{Q}\right)$$

The BCESF-DSC algorithm accomplishes the organic fusion of $Q$ CE methods by employing adjustable DS evidence theory. This approach effectively minimizes the concealed risks resulting from the high conflict of consensus results, thereby providing a novel and efficient solution to the inconsistent division issue at the CE consensus level. The details of the BCESF-DSC model are described in Algorithm 3.

## Experiments and results

In this section, the performance of three proposed algorithms (BCESF-SC, BCESF-DC, BCESF-DSC) and seven state-of-the-art CE algorithms are evaluated through experimental settings from various angles. Seven state-of-the-art comparison algorithms include entropy-based consensus clustering (ECC)^62^, weighted hierarchical agglomerative clustering (WHAC)^31^, evidence accumulation clustering (EAC)^45^, probability trajectory-based graph partitioning (PTGP)^63^, dual-granularity weighted ensemble clustering (DGWEC)^52^, ensemble clustering by propagating cluster-wise similarities (ECPCS-HC)^64^ and hybrid genetic clustering ensemble algorithm (HGCEA)^33^.

### Experimental settings and metrics

In the subsequent comparative analysis experiments, a total of fourteen datasets that are commonly used to test the performance of clustering algorithms are incorporated in this study. These include the four two-dimensional synthetic datasets^65^ (http://cs.joensuu.fi/sipu/data-sets/) and ten UCI real datasets (http://archive.ics.uci.edu/ml). Table 1 presents the basic information of the fourteen datasets in terms of serial number, name, instances, attributes, and class. To conduct a quantitative and efficient analysis of the performance disparity between the proposed algorithm and other CE algorithms, two classical metrics are adopted, namely normalized mutual information (NMI)^44^ and adjusted rand index (ARI)^66^. The larger the value of these two indicators, the closer the ensemble result is to the actual division, and the maximum value is 1.

**Table 1:** Basic information of the experimental datasets.

Serial number	Dataset name	#Instances	Attributes	#Class
Synthetic datasets				
S-1	Aggregation	788	2	7
S-2	R15	600	2	15
S-3	Compound	399	2	6
S-4	Spiral	312	2	3
UCI real datasets				
D-1	Yeast	1484	8	10
D-2	Ecoli	336	7	8
D-3	Glass	214	9	6
D-4	Iris	150	4	3
D-5	IS	2310	19	7
D-6	LR	20,000	16	26
D-7	LS	6435	36	6
D-8	SPF	1941	27	7
D-9	Wine	178	13	3
D-10	CTG	2126	21	10

The configuration of parameters for the seven comparative CE algorithms is based on the recommendations of the original literature^31,33,45,52,62–64^. To mitigate the potential impact of randomness on the fairness evaluation, the average index values (NMI and ARI) of each algorithm across 20 runs on each dataset are adopted in our study. In the experiment, the ensemble size is set as $M=20$. Under different $M$ settings, the robustness of the proposed method will be evaluated in the “The effect of ensemble size $M$ on the robustness of BCESF” section. The random initialization range of the number of clusters is set in $\left[ {2,\min \left( {\left| {C_{P} } \right|,\left\lfloor {\sqrt N } \right\rfloor } \right)} \right]$, which is explained in the “Base clustering generation process of BCESF” section. The $\theta$ in the condition ($\left|corr( .)\right|>\theta$) of high correlation attribute pair is 0.95. In addition, the value range of the number of nearest neighbors *k* in the BCESF-DSC is $\left[4, 10\right]$, and *k* within this range can more effectively represent the basic probability scenario that sample points belong to distinct clusters. Note that the BCESF-DSC approach consolidates the consensus results generated by the two newly developed CE algorithms, BCESF-SC and BCESF-DC, in the experimental settings.

### Comparative analysis of experimental results on four synthetic datasets

The primary focus of this section is to analyze the performance disparities among ten CE algorithms on four two-dimensional synthetic datasets. Table 2 presents the NMI and ARI evaluation scores for each CE algorithm on each synthetic dataset. For each dataset, the index value corresponding to the algorithm with the highest score is displayed as "score*". As illustrated in Table 2, the proposed BCESF-SC, BCESF-DC and BCESF-DSC models all achieved the outstanding performance of juxtaposing the first on S-1, S-2 and S-4 datasets. These results are followed by the ECPCS-HC and HGCEA models.

**Table 2:** NMI and ARI values of 10 clustering ensemble algorithms on synthetic datasets.

Dataset	ECC	WHAC	EAC	PTGP	DGWEC	ECPCS-HC	HGCEA	BCESF-SC	BCESF-DC	BCESF-DSC
NMI										
S-1	0.8294	0.8816	0.8733	0.8536	0.7671	0.9319	0.9263	0.9924*	0.9924*	0.9924*
S-2	0.9576	0.9249	0.9619	0.9146	0.9476	0.9706	0.9647	0.9942*	0.9942*	0.9942*
S-3	0.6727	0.7178	0.7506	0.7673	0.7646	0.8348	0.8047	0.8383	0.8329	0.8598*
S-4	0.5083	0.3438	0.5544	0.5312	0.0065	0.7937	0.8502	1*	1*	1*
ARI										
S-1	0.6533	0.8096	0.7760	0.7275	0.6755	0.9221	0.8904	0.9956*	0.9956*	0.9956*
S-2	0.8915	0.8232	0.9029	0.8305	0.8535	0.9397	0.9288	0.9928*	0.9928*	0.9928*
S-3	0.5044	0.5869	0.7359	0.7598	0.7335	0.7991	0.7788	0.8060	0.7949	0.8324*
S-4	0.4640	0.2398	0.4569	0.4165	0.0040	0.7379	0.8345	1*	1*	1*

**Figure 3:**
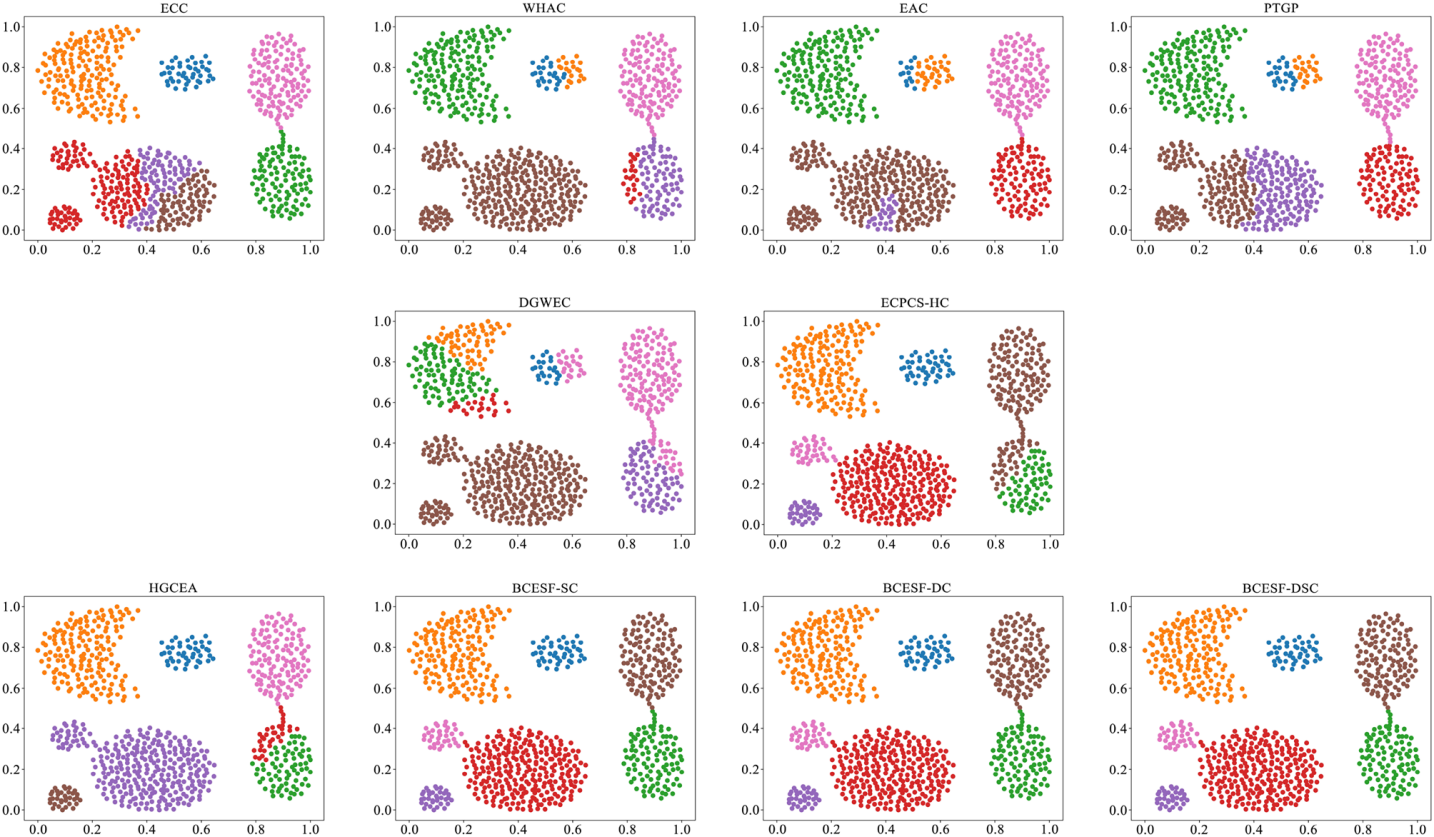
Clustering results on the synthetic dataset S-1.

**Figure 4:**
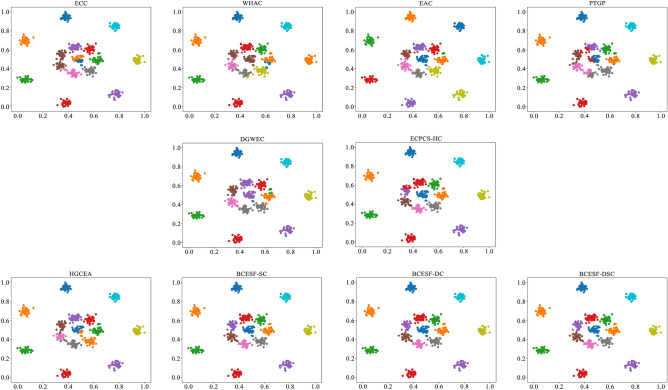
Clustering results on the synthetic dataset S-2.

**Figure 5:**
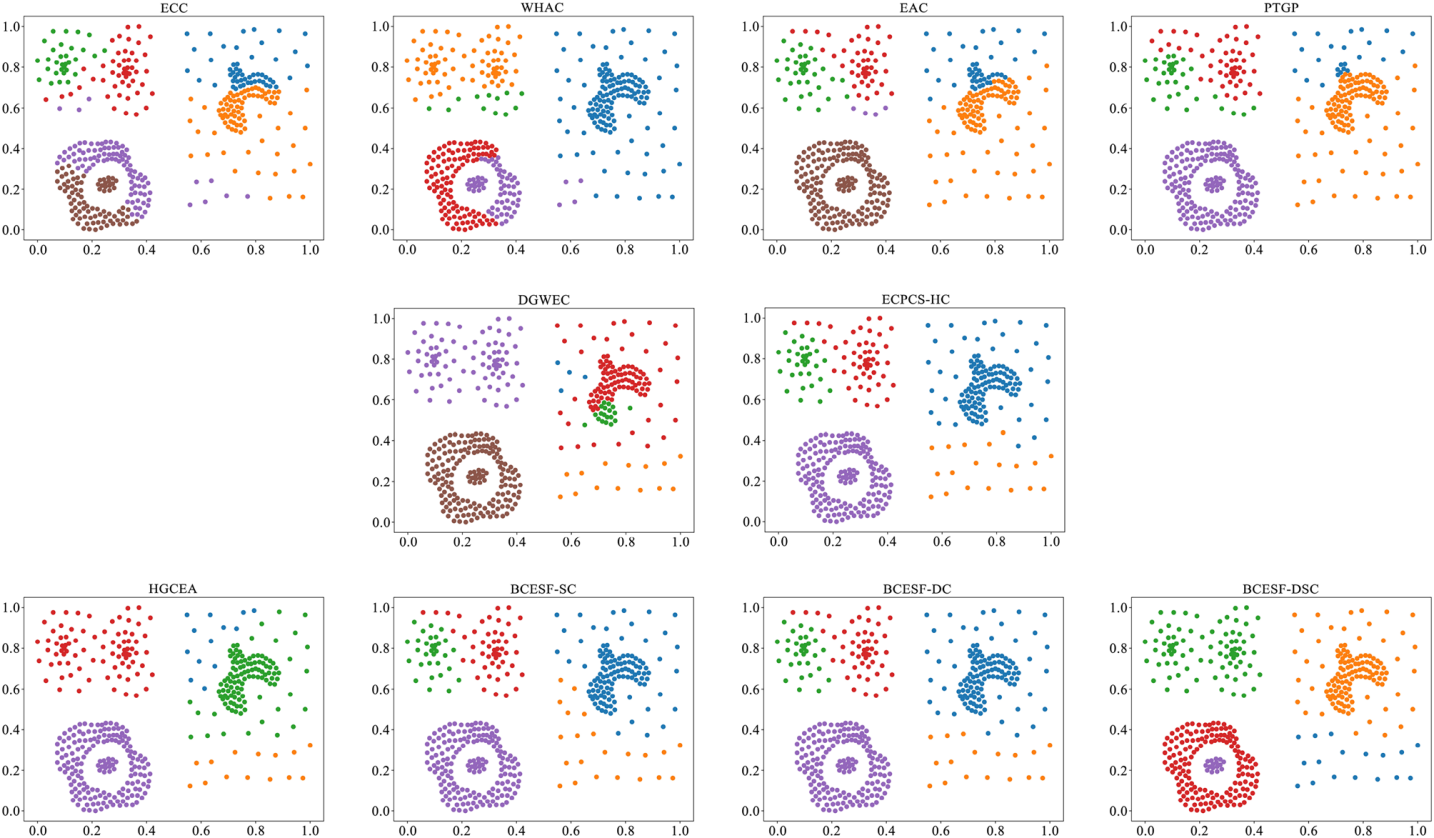
Clustering results on the synthetic dataset S-3.

**Figure 6:**
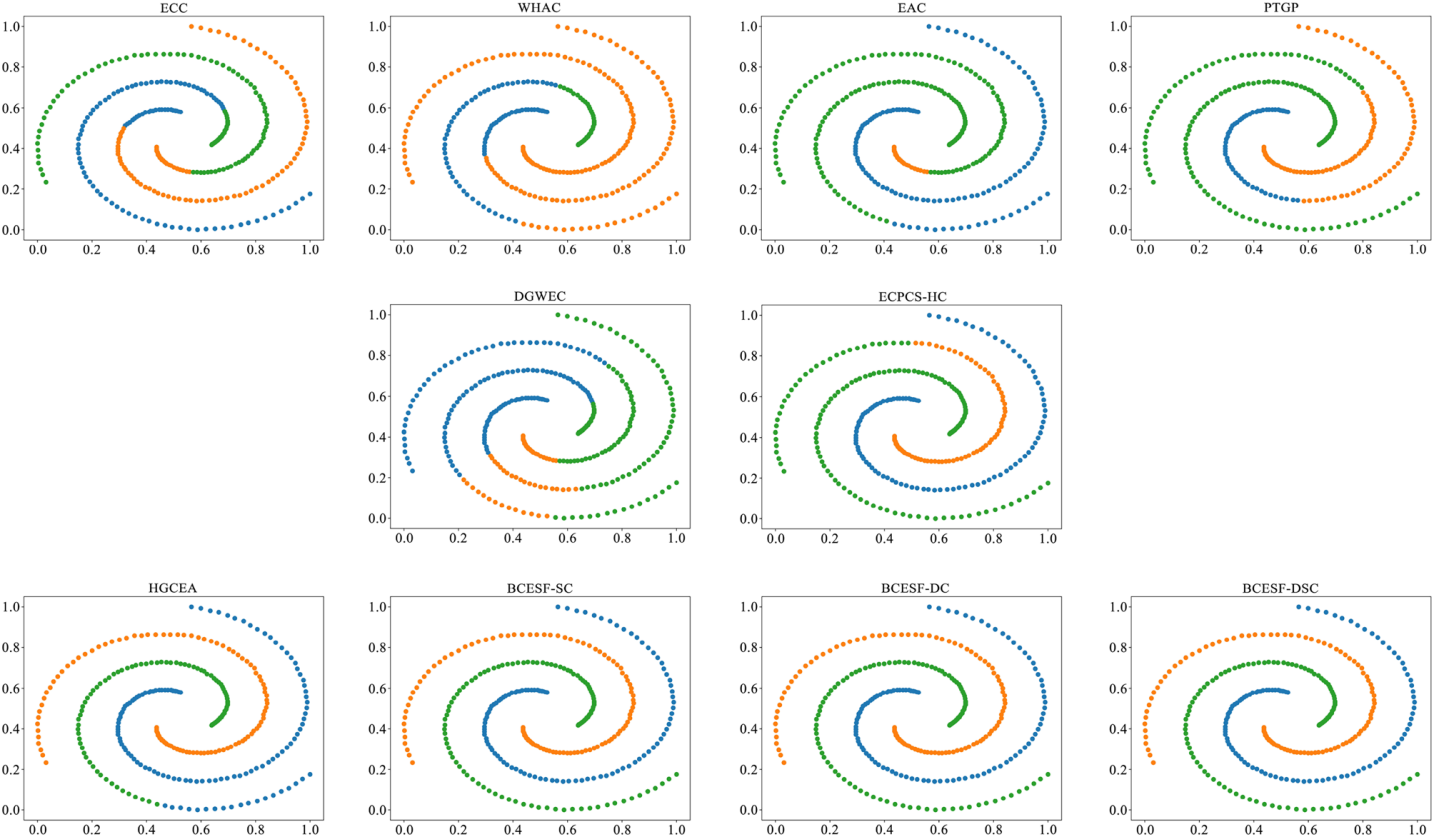
Clustering results on the synthetic dataset S-4.

However, BCESF-DSC demonstrates superior performance over BCESF-SC and BCESF-DC on the relatively complex S-3 dataset. This is attributed to the internal mechanism of BCESF-DSC, which effectively integrates the ensemble results of BCESF-SC and BCESF-DC. Furthermore, it can also be observed that when the basic CE algorithm embedded within the BCESF-DSC model achieves ideal clustering results, the potential for BCESF-DSC to further enhance the clustering effect is almost very small. In order to provide a clearer and more intuitive visualization of the clustering effects of the ten approaches on the synthetic datasets, Figs. 3, 4, 5 and 6 display their respective visualization outcomes. From Figs. 3, 4, 5 and 6, we can find that BCESF-SC, BCESF-DC and BCESF-DSC can accurately identify complex sample points at the junction of clusters, showing more outstanding conflict resolution ability than other approaches. In addition, for the S-4 dataset with manifolds distribution in Fig. 6, the three algorithms also exhibit exceptional clustering performance. This can be attributed to their incorporation of both co-occurrence relationship between sample points and the actual spatial location information, and enhance the integration quality by eliminating the redundant clustering outcomes.

### Comparative analysis of experimental results on ten UCI real datasets

**Figure 7:**
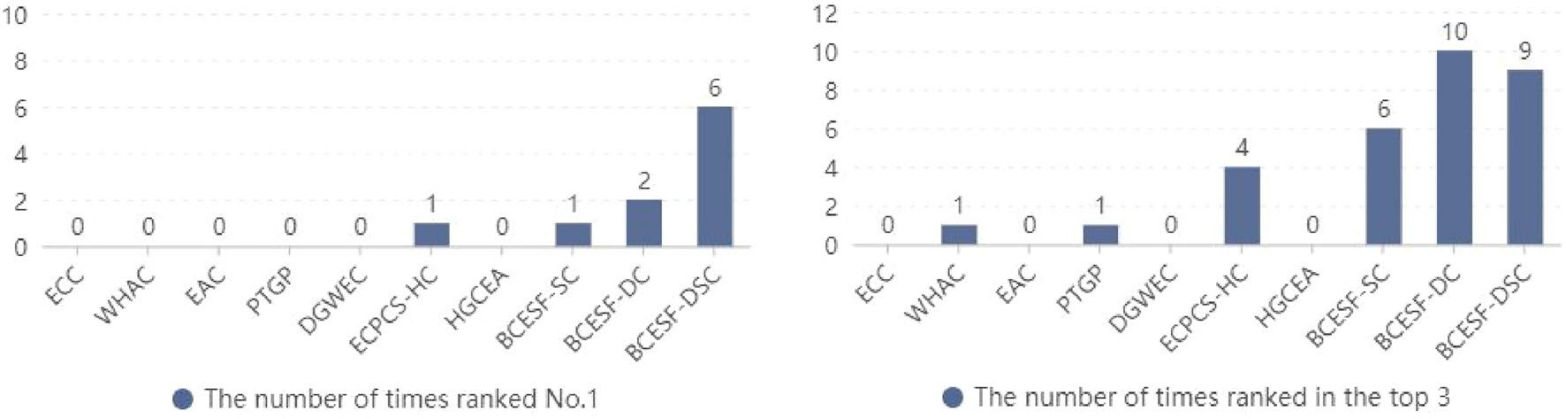
The number of times statistics that each algorithm ranks (**a**) first and (**b**) top three in NMI.

**Figure 8:**
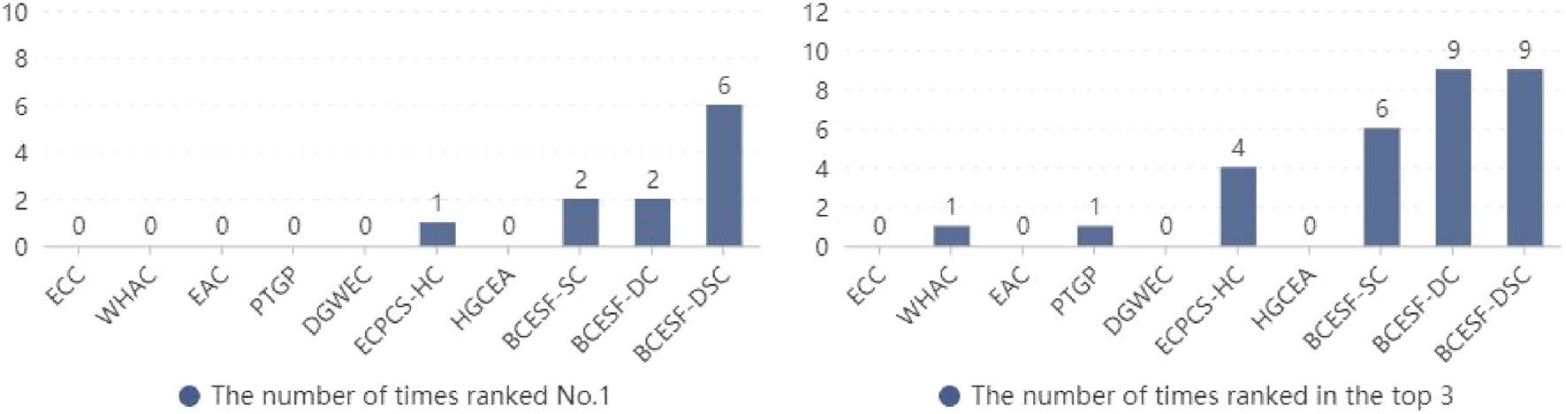
The number of times statistics that each algorithm ranks (**a**) first and (**b**) top three in ARI.

Compared to two-dimensional synthetic datasets, UCI real datasets exhibit higher feature dimensions and more complex data structures, resulting in a generally higher clustering difficulty. In this section, NMI and ARI are still adopted to quantitatively evaluate the ensemble effect of the proposed algorithms and their comparative CE algorithms. Moreover, the disparity between the performance of the algorithms is further analyzed horizontally and vertically, taking into account the size of the index value and overall ranking. In this section of the experiments, ten real datasets with varying data structures are employed as carriers. Tables 3 and 4 display the NMI and ARI index scores of the ten algorithms across each dataset, respectively. It is imperative to highlight that the index value of the algorithm that obtained the first is displayed in "score*", and the index value of the algorithms ranking among the top three is displayed in "bold" on each dataset. As a result, it is convenient to identify the number of times each algorithm has won the first and top three. Specifically, we can promptly determine the number of times that each algorithm ranks the first by looking up the "*" marks for each column in Tables 3 and 4. In a similar manner, seeking the "bold" marks to rapidly determine the number of times each algorithm gets the top three. The corresponding ranking statistics are presented in Figs. 7 and 8.

**Table 3:** NMI values of 10 clustering ensemble algorithms on UCI real datasets.

Dataset	ECC	WHAC	EAC	PTGP	DGWEC	ECPCS-HC	HGCEA	BCESF-SC	BCESF-DC	BCESF-DSC
D-1	0.1962	**0.2643**	0.2601	0.2273	0.1180	0.2710 *	0.2574	0.2188	**0.2602**	0.2579
D-2	0.5337	0.5716	0.6035	0.5316	0.6201	0.6994	0.6351	**0.7262**	**0.7252**	0.7278*
D-3	0.3023	0.3603	0.3611	**0.3617**	0.0348	0.3225	0.3061	0.3204	0.3979*	**0.3735**
D-4	0.7069	0.7852	0.7787	0.6821	0.7397	0.8756	0.8813	**0.9011**	**0.8851**	0.9144*
D-5	0.5377	0.5321	0.5310	0.5509	0.5914	0.6058	0.5914	**0.6239**	**0.6507**	0.6655*
D-6	0.3668	0.3575	0.3741	0.4022	0.4135	0.4013	0.4008	0.4546*	**0.4437**	**0.4493**
D-7	0.5542	0.6182	0.6157	0.6291	0.6332	**0.6405**	0.6026	0.6398	**0.6742**	0.6775*
D-8	0.1981	0.1407	0.1546	0.1598	0.2317	0.2864	0.2943	**0.3639**	**0.3641**	0.3648*
D-9	0.8754	0.4372	0.8662	0.8813	0.7024	**0.8926**	0.8731	**0.8926**	**0.9115**	0.9226*
D-10	0.2471	0.2546	0.2611	0.2553	0.2605	**0.2692**	0.2613	0.2689	0.3128*	**0.2786**
Avg.value	0.4518	0.4322	0.4806	0.4681	0.4345	0.5264	0.5103	0.5410	0.5625	0.5632
Avg.rank	8.6	7.6	7.1	6.9	7.05	4.05	6.05	3.75	2.2	1.7

**Table 4:** ARI values of 10 clustering ensemble algorithms on UCI real datasets.

Dataset	ECC	WHAC	EAC	PTGP	DGWEC	ECPCS-HC	HGCEA	BCESF-SC	BCESF-DC	BCESF-DSC
D-1	0.1023	**0.1657**	0.1558	0.1346	0.0193	0.1801*	0.1581	0.1235	**0.1621**	0.1355
D-2	0.3804	0.4914	0.5266	0.3652	0.5954	**0.7553**	0.5997	**0.7671**	0.7438	0.7763*
D-3	0.1623	0.2355	0.2387	**0.2401**	0.0058	0.1831	0.1657	0.1806	0.2735*	**0.2576**
D-4	0.7253	0.8101	0.7996	0.7122	0.7631	0.8984	**0.9011**	0.9222*	**0.9038**	0.9222*
D-5	0.4142	0.4078	0.3991	0.4371	0.4693	0.4786	0.4692	**0.4898**	**0.5328**	0.5451*
D-6	0.1433	0.1395	0.1513	0.1654	0.1628	0.1627	0.1598	0.1978*	**0.1667**	**0.1786**
D-7	0.4697	0.5602	0.5517	0.5468	0.5773	**0.5882**	0.5496	0.5726	**0.6085**	0.6093*
D-8	0.0445	0.0162	0.0171	0.0169	0.1225	0.1735	0.1903	**0.2163**	**0.2166**	0.2182*
D-9	0.8893	0.4657	0.8914	0.9021	0.7428	0.9126	0.8908	**0.9149**	**0.9230**	0.9344*
D-10	0.0925	0.1074	0.1121	0.1080	0.1116	**0.1203**	0.1135	0.1074	0.1489*	**0.1219**
Avg.value	0.3424	0.3400	0.3843	0.3628	0.3570	0.4453	0.4198	0.4492	0.4680	0.4699
Avg.rank	8.8	7.45	6.7	7.1	6.9	4	5.7	4.2	2.3	1.85

As illustrated in Tables 3 and 4, the NMI and ARI outcomes for BCESF-SC, BCESF-DC, BCESF-DSC and the seven comparison algorithms are reported. Specifically, the NMI values of BCESF-SC, BCESF-DC and BCESF-DSC algorithms are ranked first in 1 (D-6), 2 (D-3, D-10) and 6 (D-2, D-4, D-5, D-7, D-8, D-9) datasets, respectively. For ARI values, BCESF-SC, BCESF-DC and BCESF-DSC algorithms obtained the optimal performance in 2 (D-6, D-4), 2 (D-3, D-10) and 6 (D-2, D-4, D-5, D-7, D-8, D-9) datasets, respectively. However, ECPCS-HC secured the top position once in both indexes, with its performance trailing only the three proposed algorithms.

To provide a more comprehensive and insightful comparison of the performance of the ten algorithms, Figs. 7 and 8 count the number of times in which each algorithm ranks the first and the top three on the two metrics, respectively. The results reveal that the NMI values of BCESF-SC, BCESF-DC and BCESF-DSC are in the top three for 6 times, 10 times and 9 times, respectively. The ARI values of the three algorithms are 6 times, 9 times and 9 times are in the top three. In contrast, ECPCS-HC, which performs best among the seven comparison algorithms, won the top three on NMI and ARI for 4 times and 4 times, respectively. In addition, the average index values of each algorithm are also listed in Tables 3 and 4. By further calculation, it is indicated that the average NMI of BCESF-SC is 19.74%, 25.17%, 12.57%, 15.57%, 24.51%, 2.77% and 6.02% higher than the seven comparison algorithms (ECC, WHAC, EAC, PTGP, DGWEC, ECPCS-HC, HGCEA), respectively. The average NMI of BCESF-DC was increased by 24.50%, 30.15%, 17.04%, 20.17%, 29.46%, 6.86% and 10.23%, respectively. Compared with the seven comparison algorithms, the average NMI of BCESF-DSC increased by 24.66%, 30.31%, 17.19%, 20.32%, 29.62%, 6.99% and 10.37%, respectively. BCESF-DSC emerges as the top scorer in terms of average NMI. In a parallel manner, the average ARI improvement rate for the three methods maintains a similar advantage as the NMI improvement rate, which will not be reiterated here. As illustrated by the average rankings presented in Tables 3 and 4, BCESF-DSC and BCESF-DC, which ranked first and second, exhibit a significantly superior performance compared to other algorithms. This is followed by BCESF-SC and ECPCS-HC, which exhibit comparable results, but BCESF-SC demonstrates a marginally superior performance compared to ECPCS-HC. The overall performance of BCESF-DC and BCESF-SC is only inferior to that of BCESF-DSC, but the performance of BCESF-DC is slightly better in comparison. This discrepancy may be caused by the more robust working mechanism of the consensus strategy employed in BCESF-DC, as compared to the one used in BCESF-SC. The descending order of the average ranking performance of the remaining six algorithms is HGCEA, EAC, DGWEC, PTGP, WHAC, and ECC. All in all, the proposed BCESF-DSC, BCESF-DC, and BCESF-SC methods outperformed the other seven state-of-the-art comparison algorithms in terms of their comprehensive performance.

After conducting a comprehensive analysis from various perspectives, several significant findings have emerged. (1) The BCESF-DSC demonstrates the most superior overall performance among all the ten competing CE algorithms. This superiority can be attributed to its employment of an adjustable DS evidence theory, which enables the organic fusion of multiple ensemble algorithms. Consequently, the issue of inconsistent division at the CE consensus level is effectively addressed. (2) The BCESF-DSC, BCESF-DC, and BCESF-SC models simultaneously incorporate co-occurrence frequency and local spatial location information, which can more accurately capture the similarity relationship between sample points. (3) The processing power of the three proposed algorithms is relatively weak when applied to D-1, D-3, D-6, and D-10 datasets, and fail to achieve optimal clustering results. Additionally, the index value scores of all other compared experimental algorithms are also unsatisfactory. This may be due to the high complexity and sparsity of the dataset. In the follow-up work, we will delve into the internal mechanisms underlying such complex datasets in order to attain a more ideal partition.

### Ablation experiment

The experiments conducted in the preceding section have substantiated the significance of the key building blocks (adjustable DS evidence theory) in the BCESF-DSC model. This section continues to explore the specific utility of the “generation of base clustering results”, “backward selection strategy” and “modified relationship matrix” of the main building blocks recommended in the BCESF-SC and BCESF-DC models. To illustrate this, we consider four exemplary datasets S-2, S-4, D-2 and D-9, which comprise two synthetic datasets and two real datasets. Subsequently, employing a controlled variable method, we establish six comparative models.

- *GC-BCESF-SC (GC-BCESF-DC)* is a derivative model of BCESF-SC (BCESF-DC), which indicates that during the generation of base clustering results in the BCESF-SC (BCESF-DC) model, the random generation range for the number of clusters is changed to $\left[ {2,\left\lfloor {\sqrt N } \right\rfloor } \right]$, which is widely adopted. Additionally, the step involving filtering redundant features by Pcc is eliminated, while maintaining consistency with other implementation details of BCESF-SC (BCESF-DC).
- *NS-BCESF-SC (NS-BCESF-DC)* is another derivative model of BCESF-SC (BCESF-DC), which means that only the selection process for base clustering results is removed on the basis of BCESF-SC (BCESF-DC) model, while all other building blocks remain unchanged.
- *OI-BCESF-SC (OI-BCESF-DC)* is the third derivative model of BCESF-SC (BCESF-DC). In contrast to the BCESF-SC (BCESF-DC) model, it eliminates the utilization of the modified similarity matrix (modified distance matrix) designed in this study and solely relies on the original input matrix, while keeping all other building blocks unchanged.

The experimental outcomes are presented in Table 5. Based on the NMI and ARI index values presented in Table 5, it is evident that the methods (BCESF-SC and BCESF-DC) with the proposed building blocks have yielded superior clustering results. Moreover, the three main building blocks actually exhibit a positive role in promoting the overall performance of the model. Among them, the promoting effect of the module “generation of base clustering results” is relatively small, and the modification of “backward selection strategy” and “modified relationship matrix” modules exert a great impact on the final clustering outcomes. Furthermore, for the S-2 dataset with a relatively simple structure, the original input matrix is able to effectively capture the internal structure information. In this case, the “modified relationship matrix” building block does not provide additional enhancement. However, in terms of the degree of the overall impact, the “modified relationship matrix” exhibits greater potential for enhancing the performance of CE model. It is worth noting that once the clustering effect reaches a certain feasible degree, further improvements in clustering performance become increasingly challenging. This phenomenon becomes evident when examining the fusion effect of the DSC strategy on both BCESF-SC and BCESF-DC models as discussed in the previous section.

**Table 5:** NMI and ARI values for models with different building blocks.

Model	S-2		S-4		D-2		D-9	
	NMI	ARI	NMI	ARI	NMI	ARI	NMI	ARI
BCESF-SC	0.9942	0.9928	1	1	0.7262	0.7671	0.8926	0.9149
GC-BCESF-SC	0.9942	0.9928	1	1	0.7130	0.7461	0.8722	0.8858
NS-BCESF-SC	0.9584	0.8912	0.7937	0.7379	0.5735	0.5636	0.7625	0.7562
OI-BCESF-SC	0.9942	0.9928	0.7212	0.7080	0.5258	0.3679	0.6669	0.5580
BCESF-DC	0.9942	0.9928	1	1	0.7252	0.7438	0.9115	0.9230
GC-BCESF-DC	0.9942	0.9928	1	1	0.6853	0.7340	0.8759	0.8975
NS-BCESF-DC	0.9893	0.9857	0.8453	0.8268	0.5688	0.5497	0.7955	0.8025
OI-BCESF-DC	0.9942	0.9928	0.7937	0.7379	0.5146	0.4112	0.8473	0.8636

### The effect of ensemble size $M$ on the robustness of BCESF

**Figure 9:**
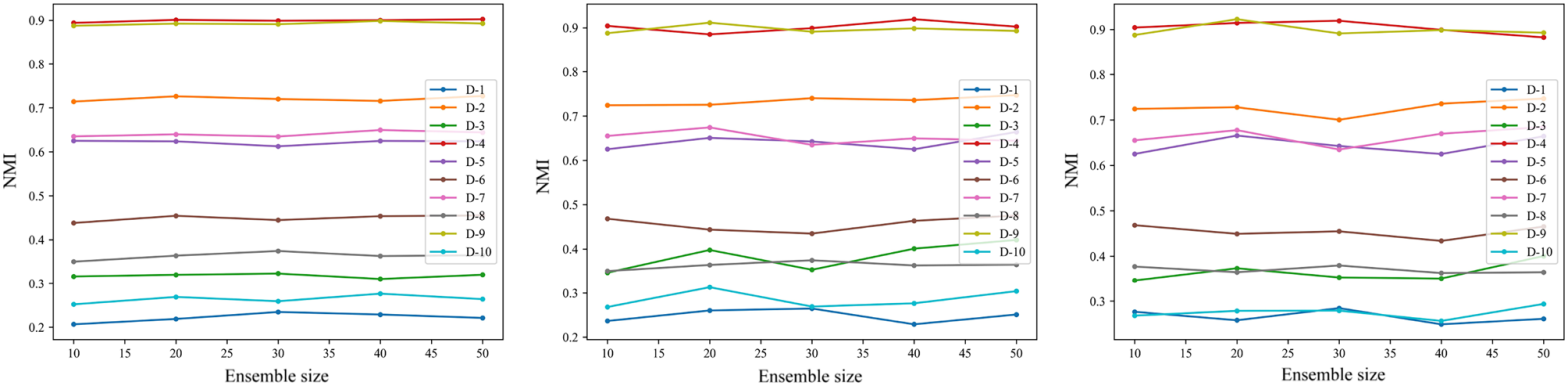
NMI values of the proposed three algorithms on ten real datasets under varying ensemble size. (**a**) BCESF-SC, (**b**) BCESF-DC, (**c**) BCESF-DSC.

**Figure 10:**
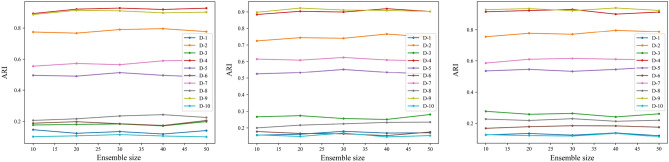
ARI values of the proposed three algorithms on ten real datasets under varying ensemble size. (**a**) BCESF-SC, (**b**) BCESF-DC, (**c**) BCESF-DSC.

The stability of the three proposed algorithms is investigated in this section, employing ten real datasets with varying ensemble sizes $M=10, 20, 30, 40, 50$, which is reflected by the fluctuations in the metrics NMI and ARI. By observing Figs. 9 and 10, it can be found that the values of NMI and ARI derived from the BCESF-SC, BCESF-DC, and BCESF-DSC algorithms tend to achieve a relatively stable state as the number of base clustering members increases, without exhibiting significant fluctuations. That is attributed to the fact that the three algorithms developed in this study establish a selection process for base clustering members, eliminating redundant members and retaining more valuable information. In consequence, despite an increase in the ensemble size $M$, the index values of the algorithms do not demonstrate a notable upward or downward trend. Consequently, only a small number of base clustering members need to be generated in our algorithm to achieve relatively ideal consensus results. Furthermore, as illustrated in Figs. 9 and 10, all three algorithms exhibit slight fluctuations on the D-3, D-7, and D-10 datasets, while they remain stable on the other seven datasets. This phenomenon might be caused by the intricate structure of the D-3, D-7, and D-10 datasets. However, this does not detract from the overall ensemble effect. Therefore, the BCESF-SC, BCESF-DC and BCESF-DSC algorithms are insensitive to the ensemble size $M$. Under various settings of $M$, the three algorithms consistently demonstrate outstanding stability.

### Running time

**Figure 11:**
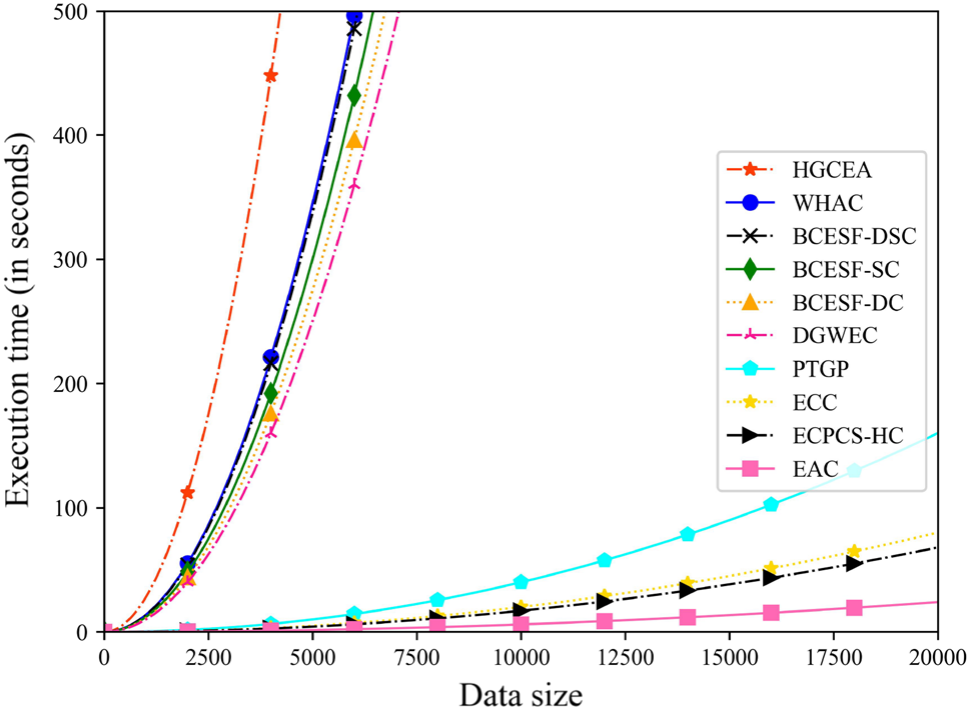
Execution time of ten clustering ensemble methods under varying data scales.

To illustrate the disparity in execution time of ten ensemble methods more graphically, a dynamic evolution approach is employed. A large-scale dataset LR with 20,000 sample points is selected to assess the efficiency of the algorithm. The ten algorithms randomly select sample points of different scales in the range of $\left[0,\mathrm{ 20,000}\right]$, and the outcomes of execution time are presented in Fig. 11. Upon examining Fig. 11, an intriguing observation can be made. According to the variation trend of execution time, the images of the ten algorithms can be distinctly categorized into two groups. One group is distributed centrally in the upper left corner of the figure and necessitates a relatively lengthy execution time, encompassing six algorithms: HGCEA, WHAC, BCESF-DSC, BCESF-SC, BCESF-DC, and DGWEC. The other group is located in the lower right corner of the figure and demands a relatively brief execution time, consisting of four algorithms: PTGP, ECC, ECPCS-HC, and EAC. The underlying reason for this phenomenon lies in the disparity of the internal execution mechanisms employed by the algorithms. Specifically, to effectively enhance the division capability of the algorithm, the six algorithms in the top-left corner either incorporate an additional iterative selection process or employ the single clustering algorithm with relatively higher complexity. Consequently, they consume more time compared to the four algorithms in the bottom-right corner. Nonetheless, the execution time of all six algorithms remains within an acceptable and reasonable range, enabling them to effectively handle large-scale datasets. In addition, the proposed three algorithms exhibit higher operational efficiency than HGCEA and WHAC, which belong to the same category. It should be noted that it does not make sense to have high execution efficiency but poor ensemble quality.

In order to conduct a more detailed analysis of the time cost details of BCESF-DSC, 5000 sample points are randomly selected from the LR datasets. Then, the BCESF-DSC is divided into four main components, including the generation process of base clustering results (T1), based-SC selection process (T2), based-DC selection process (T3), and the fusion process of DSC (T4), so as to discuss the specific time spent on each component. The experimental results are presented in Table 6. As observed from Table 6, T1 accounts for most of the execution time of BCESF-DSC. The fusion process of DSC is remarkably fast. It is worth noting that the BCESF-DSC takes the ensemble results of BCESF-SC and BCESF-DC models as the fusion object in the experiment. However, the complexity of the single clustering algorithms used in both models is relatively high. In other words, the execution time of BCESF-DSC takes into account all the time costs involved, which consequently results in higher time expenditure. In fact, if other fast CE models are used as fusion objects for BCESF-DSC in practical applications, less time can be spent.

**Table 6:** The time cost of the BCESF-DSC algorithm on each component.

Each component	T1	T2	T3	T4
Execution time	280.9 s	48 s	55.9 s	2.9
Total time	387.7 s			

## Conclusions

At the dual-level of base clustering consensus and CE consensus, an extended CE algorithm with three consensus strategies, called BCESF-DSC, is successfully proposed, which has the best overall performance in the experiment. First of all, a backward clustering ensemble selection framework is designed, in which the selection strategy can adaptively pick out the optimal member combination without preset parameters. Second, at the base clustering consensus level, the SC and DC consensus strategies profoundly mine the interrelation between co-occurrence frequency and actual spatial location information, thereby capturing the co-occurrence relationship of sample pairs more comprehensively. Among them, the SC strategy employs the modified similarity matrix, derived from the distance matrix, as the crucial input for the ultimate consensus result. DC modifies the distance matrix using the similarity matrix to complete the final clustering. Furthermore, the third consensus strategy, DSC, employs an adjustable DS evidence theory to effectively and dynamically fuse multiple ensemble algorithms. This fundamentally resolves the conflict issue of inconsistent division at the CE consensus level with a broader perspective. Finally, the effectiveness of the proposed algorithm is further corroborated by multi-angle comparative analysis experiments. It is worth noting that the indirectly proposed CE algorithms, BCESF-SC and BCESF-DC, can also be utilized effectively and independently.

Although this study presents three novel and potent strategies for the field of clustering research, the algorithm may suffer from unbearable time cost when addressing huge-scale clustering tasks. Consequently, our future research endeavors will focus on the development of ingenious sparse techniques to further enhance the algorithm's efficiency.

## Data Availability

All data generated or analysed during this study are included in this article.
